# Esophageal Cancer Genomics in Africa: Recommendations for Future Research

**DOI:** 10.3389/fgene.2022.864575

**Published:** 2022-03-25

**Authors:** Hannah Simba, Gerard Tromp, Vikash Sewram, Christopher G Mathew, Wenlong C. Chen, Helena Kuivaniemi

**Affiliations:** ^1^ African Cancer Institute, Department of Global Health, Faculty of Medicine and Health Sciences, Stellenbosch University, Cape Town, South Africa; ^2^ Environment and Lifestyle Epidemiology Branch, International Agency for Research on Cancer (IARC/WHO), Lyon, France; ^3^ Division of Molecular Biology and Human Genetics, Department of Biomedical Sciences, Faculty of Medicine and Health Sciences, Stellenbosch University, Cape Town, South Africa; ^4^ DSI–NRF Centre of Excellence for Biomedical Tuberculosis Research, Stellenbosch University, Cape Town, South Africa; ^5^ South African Medical Research Council Centre for Tuberculosis Research, Stellenbosch University, Cape Town, South Africa; ^6^ Bioinformatics Unit, South African Tuberculosis Bioinformatics Initiative, Stellenbosch University, Cape Town, South Africa; ^7^ Centre for Bioinformatics and Computational Biology, Stellenbosch University, Stellenbosch, South Africa; ^8^ Sydney Brenner Institute for Molecular Bioscience, Faculty of Health Sciences, University of the Witwatersrand, Johannesburg, South Africa; ^9^ Division of Human Genetics, National Health Laboratory Service and School of Pathology, Faculty of Health Sciences, University of the Witwatersrand, Johannesburg, South Africa; ^10^ National Cancer Registry, National Health Laboratory Service, Johannesburg, South Africa

**Keywords:** esophageal cancer, esophageal squamous cell carcinoma, genomics, genomic medicine, African esophageal cancer corridor

## Introduction

Esophageal cancer (EC) is an aggressive malignancy and a major health burden documented as the sixth most common cause of cancer mortality worldwide (Bray et al., 2018). Over 80% of EC cases and deaths are reported in developing countries, where the esophageal squamous cell carcinoma (ESCC) subtype is more common, compared to the adenocarcinoma (EAC) subtype (Bray et al., 2018). EC has a peculiar geographical distribution, with high incidence rates reported in Asia and the African ESCC corridor (Abnet et al., 2017; McCormack et al., 2017; Bray et al., 2018). Malawi has the highest ESCC incidence rate globally for both men and women, followed by Kenya and Zimbabwe in Africa (Bray et al., 2018). In South Africa, ESCC is the 10th most common cancer for men and the 11th most common cancer for women (National Cancer Registry, 2019), and has the 10th highest incidence of ESCC in Africa (Bray et al., 2018). Incidence rates are, however, disproportionately higher in the Eastern Cape Province, where it is the most common cancer for men, and the second most common cancer for women (Somdyala et al., 2015). Due to a lack of ESCC early detection markers, late diagnosis and poor prognosis are the norm. Additionally, distinct ESCC molecular subtypes have not been identified, which could provide opportunities for targeted and novel therapies.

**FIGURE 1:**
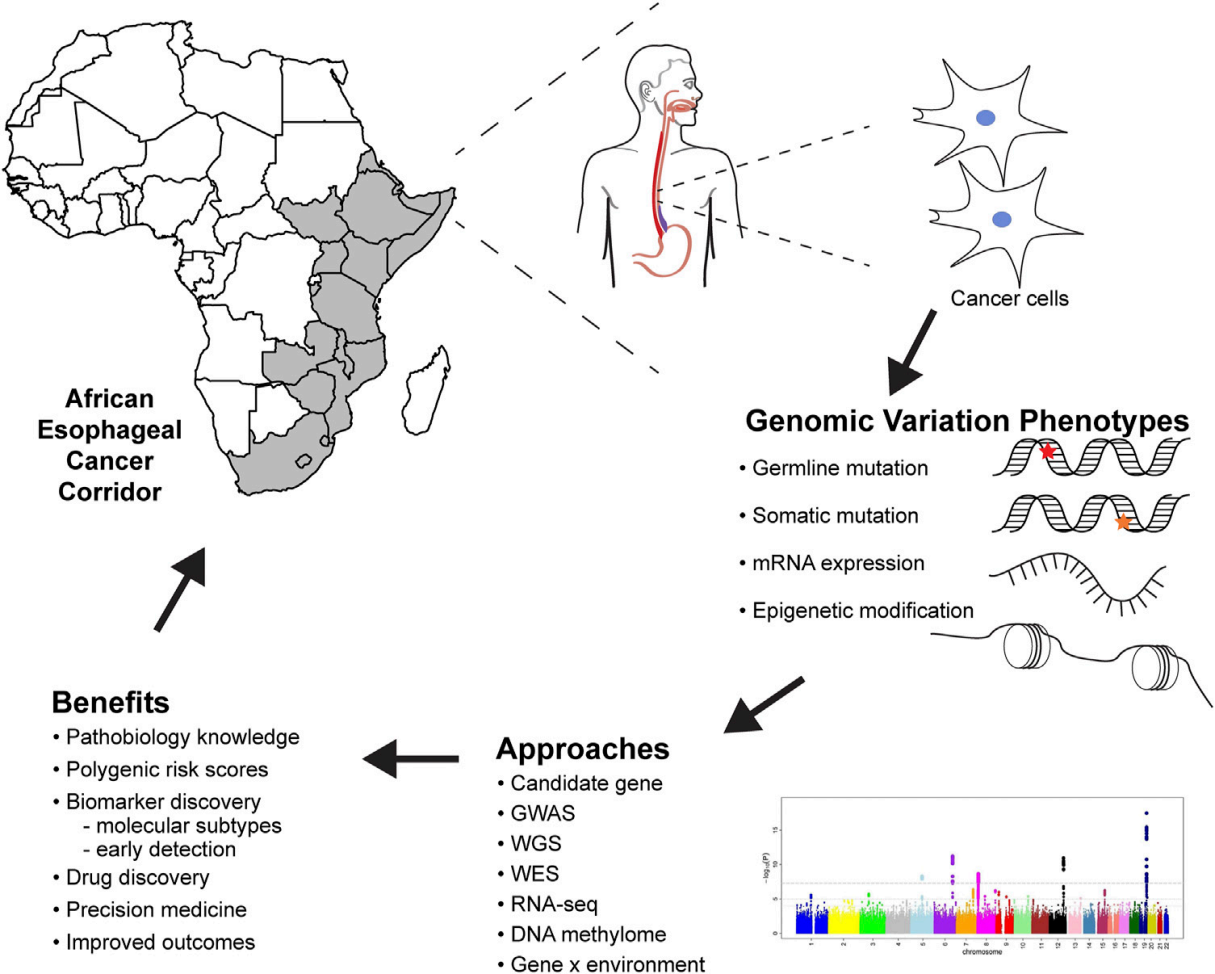
Future of esophageal squamous cell carcinoma genomic research in Africa, methods, analysis, and benefits to genomic medicine. GWAS, genome-wide association studies; WGS, whole-genome sequencing; WES, whole-exome sequencing; RNA-seq, RNA sequencing.

The African ESCC corridor (Figure 1), which spans from the eastern to the southern part of Africa is characterized by high incidence rates, young age at presentation, delayed presentation, as well as poor outcomes and survival (Van Loon et al., 2018; Asombang et al., 2019). Risk factors associated with ESCC in high-risk areas include tobacco smoking, alcohol consumption, polycyclic aromatic hydrocarbon exposure, poor diet, hot beverages, poor oral hygiene, microbiome, and genetic factors (Asombang et al., 2019; Chetwood et al., 2019; Simba et al., 2019; Simba, 2021).

### Limited Number of Genomic Studies on ESCC in Africa

There is an apparent lack of genomic studies on ESCC on African populations, therefore the genetic etiology is poorly understood and implementation of genomic medicine for ESCC remains elusive. The striking geographical distribution of ESCC suggests that ESCC etiology is multifactorial, with shared and locally relevant environmental and genetic risk factors. There are several mutational signatures in ESCC that have been linked to environmental exposures, including tobacco smoking and alcohol consumption (Talukdar et al., 2013; Matejcic and Parker, 2015). It is, therefore, important to incorporate environmental and lifestyle risk factors when investigating genetic factors. Identification and quantification of these gene-environment (GxE) interactions provides a platform for targeted interventions and may explain the high incidence and mortality rates in Africa. In this opinion piece, we summarize the current state of ESCC genomic research in Africa, which include candidate gene studies, whole-exome studies and genome-wide association studies (GWAS). The first genome-wide methylation profiling of ESCC, with 327 tissues from nine African, Asian and South American populations, identified novel DNA methylation events in ESCC tumors (Talukdar et al., 2021). Importantly, there is a lack of multi-omics studies.

### Candidate Gene Studies in African ESCC

We recently performed a systematic review of candidate gene studies carried out for ESCC in African populations (Simba et al., 2019). Studies were done on South African, Malawian and Kenyan populations only (Simba et al., 2019). Only one exome sequencing study from Malawi on ESCC tumors was found (Liu et al., 2016). In the germline, 25 SNPs were reported to be associated with ESCC in 20 different genes, and 22 genes had somatic mutations (Simba et al., 2019)*.* These studies had several limitations: 1) small sample sizes leading to poor statistical power; 2) no cohort studies; and 3) no standardized methods of data collection, reporting, or analysis. In addition, the studies did not correct for population structure in cases and controls, which is particularly relevant in African populations known for high levels of genomic diversity (Choudhury et al., 2020).

### Genome-Wide Association Studies in African ESCC are Needed

All GWAS for ESCC published to date were conducted in Asian or European populations (Cui et al., 2009; Wang et al., 2010; McKay et al., 2011; Wu et al., 2012; Wu et al., 2013; Wu et al., 2014; Abnet et al., 2017). These studies identified several associated loci, including *PLCE1*, *RUNX1* and *CHEK2*. However, the transferability and replication of these loci in African populations were limited, even in a well-powered study (Chen et al., 2019). Thus there is a need for ESCC GWAS studies in populations from high-risk regions of sub-Saharan Africa. GWAS are currently in progress in the ERICA-SA study (https://www.samrc.ac.za/intramural-research-units/evolving-risk-factors-cancers-african-populations-erica-sa), Johannesburg Cancer study (Chen et al., 2020), and the African Esophageal Cancer Consortium (AfrECC) (Van Loon et al., 2018). These studies are likely to provide a much clearer understanding of the genetic etiology of African ESCC. GWAS data can also be used to develop polygenic risk scores for stratifying disease risk (Fritsche et al., 2021).

### Somatic Mutations in Tumor Tissue From African ESCC Patients

Genomic analysis of tumor tissues by DNA and RNA sequencing in large-scale global projects such as the Cancer Genome Atlas (TCGA) (The Cancer Genome Atlas Research Network, 2017) has led to a huge increase in knowledge of the genes and somatic mutations which drive tumor development. This in turn has fueled major progress in the development of cancer therapies that target the molecular pathways important for tumor development. No samples from African ESCC cases were included in the TCGA project. Information on the genomic profiles of African cancers is, however, very limited. In ESCC, whole-exome and RNA sequencing of tumors from 59 Malawian patients (Liu et al., 2016) observed similar genetic aberrations to those reported in Asian and North American patients and included mutations of well-established driver genes such as *TP53, CDKN2A, NFE2L2, CHEK2, NOTCH1, FAT1*, and *FBXW7*. Analyses also detected signatures associated with aging and cytidine deaminase (APOBEC) activity but, surprisingly, not of tobacco smoke.

A recent study (Moody et al., 2021) investigated mutational signatures in 552 ESCC patients from high-incidence regions (Iran, Kenya, Tanzania, China and Malawi) including three African countries, and low-incidence regions (Brazil, Japan and the UK) using whole-genome sequencing. Similar mutational profiles across all countries were found. Specific mutational signatures and ESCC risk factors were detected for tobacco, alcohol, opium and germline variants, and also highlighted APOBEC activation as an important step in tumor development. No evidence of an unknown exogenous mutagen associated with a mutational signature, which could explain ESCC variation in incidence, was found.

## Discussion

### Benefits of Genomic Data in ESCC Research

Genomic studies provide critical information about the pathobiology of diseases, which improves our understanding of the risk and heritability of ESCC, risk prediction for populations and individuals, and contributes to cancer prevention (Figure 1). ESCC genomic research must follow rigorous guidelines to ensure reproducibility and reliability of results. Prioritization of ESCC genomic medicine in African populations will help elucidate the genetic etiology of ESCC, giving insights on variants, biological mechanisms, and the GxE interactions associated with ESCC development. This information will facilitate developing algorithms for predicting ESCC prognosis and survival of the patients.

Genomics can be used as a tool to address health disparities in cancer (Balogun and Olopade, 2021). There are differences in ESCC incidence and mortality between European and African populations. Whilst inadequate health care systems, lack of access, and poor quality of care have an obvious role, genomics can also be used to address these gaps in incidence and mortality. Genomic research can identify variants, pathways and biomarkers associated with increased risk and mortality, to be used in precision medicine (Figure 1). Ultimately, ESCC genomic research in African populations should not only contribute to understanding the etiology but also generate evidence that can be translated to prevention and therapeutics.

A recent study (Choudhury et al., 2020) explored human migration and the breadth of genomic diversity in 426 African individuals from 50 ethnolinguistic groups. Over three million previously undescribed single nucleotide variants were identified. The authors recommended “*broader characterization of the genomic diversity of African individuals to understand human ancestry and improve health*” (Choudhury et al., 2020). It remains to be determined if ancestral events, migration, the admixture of populations, and adaptation to exposures in the African ESCC corridor play a role in the demographic and geographic aspects of ESCC.

The Pan-Cancer Analysis of Whole Genomes Consortium performed the most comprehensive meta-analysis of cancer genomes to date, using 2,658 tumors and 38 tissues (Cieslik and Chinnaiyan, 2020). Of these, 98 were EAC tumors, but none were ESCC samples. The study assessed key aspects of cancer genomics, i.e., cancer drivers (Campbell et al., 2020), non-coding changes (Rheinbay et al., 2020), mutational signatures (Li et al., 2020), structural variants (Alexandrov et al., 2020), cancer evolution (Gerstung et al., 2020), and RNA alterations (Calabrese et al., 2020). However, none of the investigators were from Africa. Genomic analysis of substantial numbers of African ESCC patients together with detailed epidemiological data is needed to further explore the origins of African ESCC.

### Barriers to ESCC Genomics Research in Africa

The lack of investment in genomic medicine in Africa has led to most genomic medicine knowledge being founded on genomes of European ancestry, despite African populations displaying higher levels of genetic diversity. A major barrier to implementing genomic research in Africa is inadequate infrastructure including poorly equipped facilities, erratic power supply, inadequate biotechnology and information technology infrastructure as well as the high cost of genomics tools and the costs associated with implementing genomic medicine (Adebamowo et al., 2018; Munung et al., 2018). These aspects further impact biospecimen collection, transportation and storage, which is pivotal in planning and conducting genomic studies. In Africa, the ethical aspects surrounding genomics research are also challenging and obtaining informed consent is compounded by language barriers and low literacy levels.

### Recommendations for Future ESCC Studies and Furthering Genomics Research in Africa

African genomes harbor the most genetic diversity and variation, and yet are the least genetically characterized. This means that genetic variants of medical relevance remain unknown (Choudhury et al., 2020). For ESCC this impedes progress on applying genomics in understanding etiology, tailored screening and therapeutic interventions, promoting health equity and ultimately reducing the burden of ESCC in the African ESCC corridor. To fully understand the etiology of ESCC and provide tools for genomic medicine, ESCC research in Africa should: 1) follow a multidisciplinary approach to study interactions between genomic, environmental and lifestyle factors; 2) foster collaborations and data sharing to accelerate progress; 3) use standardized methods for analysis and reporting; 4) use larger study samples to adequately detect GxE interactions; and 5) control for population stratification in admixed populations. Additionally, openness and precision in reporting of methods are needed to improve reproducibility. In a cancer biology study aimed at replicating 193 experiments from 53 high-impact papers, none of the published studies had sufficient information for repeating the experiments (Errington et al., 2021). For genetic association studies, the STrengthening the REporting of Genetic Association studies (STREGA) statement (Little et al., 2009) should be used as a checklist to assess quality of reporting and methods.

Furthering genomic medicine in Africa requires leveraging existing infrastructure and learning from the extensive experience of current genomic medicine implementations in other countries (AESA, 2020). This requires significant infrastructure, including access to clinical facilities and high-throughput genotyping and sequencing facilities. Creating a unified ESCC genomics research hub in Africa will require standardized sample collection and well-managed biorepositories with the capacity to store and manage biospecimens. Strong information technology infrastructure is needed that is capable of managing, storing and analyzing big data (Wonkam and Mayosi, 2014). In addition, capacity building is needed to create a critical mass of bioinformaticians, and provide genomic medicine training programs for healthcare professionals.

## Conclusion

Genomics is an invaluable approach in providing unbiased information about the pathogenesis of ESCC. The information could be used to predict risk, screen asymptomatic individuals, diagnose more accurately and develop targeted treatments. We are still far from being able to implement genomic medicine for ESCC in Africa since genomic information on African ESCC patients is very limited.

## References

[B1] Abnet C. C. Arnold M. Wei W.-Q. (2018). Epidemiology of Esophageal Squamous Cell Carcinoma. Gastroenterology 154 (2), 360–373. 10.1053/j.gastro.2017.08.023 28823862 PMC5836473

[B2] Adebamowo S. N. Francis V. Tambo E. Diallo S. H. Landouré G. Nembaware V. (2018). Implementation of Genomics Research in Africa: Challenges and Recommendations. Glob. Health Action. 11 (1), 1419033. 10.1080/16549716.2017.1419033 29336236 PMC5769805

[B3] AESA (2020). “A Framework for the Implementation of Genomic Medicine for Public Health in Africa,” in Alliance for Accelerating Excellence in Science in Africa (Nairobi).

[B4] Alexandrov L. B. Kim J. Kim J. Haradhvala N. J. Huang M. N. Tian Ng A. W. (2020). The Repertoire of Mutational Signatures in Human Cancer. Nature 578 (7793), 94–101. 10.1038/s41586-020-1943-3 32025018 PMC7054213

[B5] Asombang A. W. Chishinga N. Nkhoma A. Chipaila J. Nsokolo B. Manda-Mapalo M. (2019). Systematic Review of Esophageal Cancer in Africa: Epidemiology, Risk Factors, Management and Outcomes. Wjg 25 (31), 4512–4533. 10.3748/wjg.v25.i31.4512 31496629 PMC6710188

[B6] Balogun O. D. Olopade O. I. (2021). Addressing Health Disparities in Cancer with Genomics. Nat. Rev. Genet. 22 (10), 621–622. 10.1038/s41576-021-00390-4 34244675 PMC8267506

[B7] Bray F. Ferlay J. Soerjomataram I. Siegel R. L. Torre L. A. Jemal A. (2018). Global Cancer Statistics 2018: GLOBOCAN Estimates of Incidence and Mortality Worldwide for 36 Cancers in 185 Countries. CA: a Cancer J. clinicians 68 (6), 394–424. 10.3322/caac.21492 30207593

[B8] Calabrese C. Davidson N. R. Davidson N. R. Demircioğlu D. Fonseca N. A. He Y. (2020). Genomic Basis for RNA Alterations in Cancer. Nature 578 (7793), 129–136. 10.1038/s41586-020-1970-0 32025019 PMC7054216

[B9] Campbell P. J. Getz G. Korbel J. O. Stuart J. M. Jennings J. L. Stein L. D. (2020). Pan-cancer Analysis of Whole Genomes. Nature 578 (7793), 82–93. 10.1038/s41586-020-1969-6 32025007 PMC7025898

[B10] Chen W. C. Bye H. Matejcic M. Amar A. Govender D. Khew Y. W. (2019). Association of Genetic Variants in CHEK2 with Oesophageal Squamous Cell Carcinoma in the South African Black Population. Carcinogenesis 40 (4), 513–520. 10.1093/carcin/bgz026 30753320 PMC6556703

[B11] Chen W. C. Singh E. Muchengeti M. Bradshaw D. Mathew C. G. Babb de Villiers C. (2020). Johannesburg Cancer Study (JCS): Contribution to Knowledge and Opportunities Arising from 20 Years of Data Collection in an African Setting. Cancer Epidemiol. 65, 101701. 10.1016/j.canep.2020.101701 32169796

[B12] Chetwood J. D. Garg P. Finch P. Gordon M. (2019). Systematic Review: the Etiology of Esophageal Squamous Cell Carcinoma in Low-Income Settings. Expert Rev. Gastroenterol. Hepatol. 13 (1), 71–88. 10.1080/17474124.2019.1543024 30791842

[B13] Choudhury A. Aron S. Botigué L. R. Sengupta D. Botha G. Bensellak T. (2020). High-depth African Genomes Inform Human Migration and Health. Nature 586 (7831), 741–748. 10.1038/s41586-020-2859-7 33116287 PMC7759466

[B14] Cieslik M. Chinnaiyan A. M. (2020). Global Genomics Project Unravels Cancer's Complexity at Unprecedented Scale. Nature 578 (7793), 39–40. 10.1038/d41586-020-00213-2 32025004

[B15] Cui R. Kamatani Y. Takahashi A. Usami M. Hosono N. Kawaguchi T. (2009). Functional Variants in ADH1B and ALDH2 Coupled with Alcohol and Smoking Synergistically Enhance Esophageal Cancer Risk. Gastroenterology 137 (5), 1768–1775. 10.1053/j.gastro.2009.07.070 19698717

[B16] Errington T. M. Denis A. Perfito N. Iorns E. Nosek B. A. (2021). Challenges for Assessing Replicability in Preclinical Cancer Biology. Elife 10. 10.7554/eLife.67995 PMC8651289 34874008

[B17] Fritsche L. G. Ma Y. Zhang D. Salvatore M. Lee S. Zhou X. (2021). On Cross-Ancestry Cancer Polygenic Risk Scores. Plos Genet. 17 (9), e1009670. 10.1371/journal.pgen.1009670 34529658 PMC8445431

[B18] Gerstung M. Jolly C. Jolly C. Leshchiner I. Dentro S. C. Gonzalez S. (2020). The Evolutionary History of 2,658 Cancers. Nature 578 (7793), 122–128. 10.1038/s41586-019-1907-7 32025013 PMC7054212

[B19] Li Y. Roberts N. D. Roberts N. D. Wala J. A. Shapira O. Schumacher S. E. (2020). Patterns of Somatic Structural Variation in Human Cancer Genomes. Nature 578 (7793), 112–121. 10.1038/s41586-019-1913-9 32025012 PMC7025897

[B20] Little J. Higgins J. P. T. Ioannidis J. P. A. Moher D. Gagnon F. von Elm E. (2009). Strengthening the Reporting of Genetic Association Studies (STREGA)-an Extension of the Strengthening the Reporting of Observational Studies in Epidemiology (STROBE) Statement. J. Clin. Epidemiol. 62 (6), 597–608. e594. 10.1016/j.jclinepi.2008.12.004 19217256

[B21] Liu W. Snell J. M. Jeck W. R. Hoadley K. A. Wilkerson M. D. Parker J. S. (2016). Subtyping Sub-saharan Esophageal Squamous Cell Carcinoma by Comprehensive Molecular Analysis. JCI Insight 1 (16), e88755. 10.1172/jci.insight.88755 27734031 PMC5053149

[B22] Matejcic M. Iqbal Parker M. (2015). Gene-environment Interactions in Esophageal Cancer. Crit. Rev. Clin. Lab. Sci. 52 (5), 211–231. 10.3109/10408363.2015.1020358 26220475

[B23] McCormack V. A. Menya D. Munishi M. O. Dzamalala C. Gasmelseed N. Leon Roux M. (2017). Informing Etiologic Research Priorities for Squamous Cell Esophageal Cancer in Africa: A Review of Setting-specific Exposures to Known and Putative Risk Factors. Int. J. Cancer 140 (2), 259–271. 10.1002/ijc.30292 27466161 PMC5763498

[B24] McKay J. D. Truong T. Gaborieau V. Chabrier A. Chuang S. C. Byrnes G. (2011). A Genome-wide Association Study of Upper Aerodigestive Tract Cancers Conducted within the INHANCE Consortium. Plos Genet. 7 (3), e1001333. 10.1371/journal.pgen.1001333 21437268 PMC3060072

[B25] Moody S. Senkin S. Islam S. M. A. Wang J. Nasrollahzadeh D. Cortez Cardoso Penha R. (2021). Mutational Signatures in Esophageal Squamous Cell Carcinoma from Eight Countries with Varying Incidence. Nat. Genet. 53 (11), 1553–1563. 10.1038/s41588-021-00928-6 34663923

[B26] Munung N. S. Mayosi B. M. de Vries J. (2018). Genomics Research in Africa and its Impact on Global Health: Insights from African Researchers. Glob. Health Epidemiol. 3, e12. 10.1017/gheg.2018.3 PMC6152488 30263136

[B27] National Cancer Registry (2019). Cancer in South Africa. Johannesburg: National Health Laboratory Services. (NCR).

[B28] Rheinbay E. Nielsen M. M. Nielsen M. M. Abascal F. Wala J. A. Shapira O. (2020). Analyses of Non-coding Somatic Drivers in 2,658 Cancer Whole Genomes. Nature 578 (7793), 102–111. 10.1038/s41586-020-1965-x 32025015 PMC7054214

[B29] Simba H. Kuivaniemi H. Lutje V. Tromp G. Sewram V. (2019). Systematic Review of Genetic Factors in the Etiology of Esophageal Squamous Cell Carcinoma in African Populations. Front. Genet. 10, 642. 10.3389/fgene.2019.00642 31428123 PMC6687768

[B30] Simba H. (2021). The Role of Genetic and Environmental Factors in the Aetiology of Esophageal CancerPhD (Public Health) Stellenbosch University. https://scholar.sun.ac.za/handle/10019.1/123653.

[B31] Somdyala N. I. M. Parkin D. M. Sithole N. Bradshaw D. (2015). Trends in Cancer Incidence in Rural Eastern Cape Province; South Africa, 1998-2012. Int. J. Cancer 136 (5), E470–E474. 10.1002/ijc.29224 25236502

[B32] Talukdar F. R. Ghosh S. K. Laskar R. S. Mondal R. (2013). Epigenetic, Genetic and Environmental Interactions in Esophageal Squamous Cell Carcinoma from Northeast India. PLoS One 8 (4), e60996. 10.1371/journal.pone.0060996 23596512 PMC3626640

[B33] Talukdar F. R. Soares Lima S. C. Khoueiry R. Laskar R. S. Cuenin C. Sorroche B. P. (2021). Genome-Wide DNA Methylation Profiling of Esophageal Squamous Cell Carcinoma from Global High-Incidence Regions Identifies Crucial Genes and Potential Cancer Markers. Cancer Res. 81 (10), 2612–2624. 10.1158/0008-5472.CAN-20-3445 33741694

[B34] The Cancer Genome Atlas Research Network (2017). Integrated Genomic Characterization of Oesophageal Carcinoma. Nature 541 (7636), 169–175. 10.1038/nature20805 28052061 PMC5651175

[B35] Van Loon K. Mwachiro M. M. Abnet C. C. Akoko L. Assefa M. Burgert S. L. (2018). The African Esophageal Cancer Consortium: A Call to Action. Jgo, 1–9. 10.1200/JGO.17.00163 PMC6223465 30241229

[B36] Wang L. D. Zhou F. Y. Li X. M. Sun L. D. Song X. Jin Y. (2010). Genome-wide Association Study of Esophageal Squamous Cell Carcinoma in Chinese Subjects Identifies Susceptibility Loci at PLCE1 and C20orf54. Nat. Genet. 42 (9), 759–763. 10.1038/ng.648 20729853

[B37] Wonkam A. Mayosi B. M. (2014). Genomic Medicine in Africa: Promise, Problems and Prospects. Genome Med. 6 (2), 11. 10.1186/gm528 25031612 PMC3979013

[B38] Wu C. Wang Z. Song X. Feng X. S. Abnet C. C. He J. (2014). Joint Analysis of Three Genome-wide Association Studies of Esophageal Squamous Cell Carcinoma in Chinese Populations. Nat. Genet. 46 (9), 1001–1006. 10.1038/ng.3064 25129146 PMC4212832

[B39] Wu C. Kraft P. Zhai K. Chang J. Wang Z. Li Y. (2012). Genome-wide Association Analyses of Esophageal Squamous Cell Carcinoma in Chinese Identify Multiple Susceptibility Loci and Gene-Environment Interactions. Nat. Genet. 44 (10), 1090–1097. 10.1038/ng.2411 22960999

[B40] Wu C. Li D. Jia W. Hu Z. Zhou Y. Yu D. (2013). Genome-wide Association Study Identifies Common Variants in SLC39A6 Associated with Length of Survival in Esophageal Squamous-Cell Carcinoma. Nat. Genet. 45 (6), 632–638. 10.1038/ng.2638 23644492

